# Large thigh liposarcoma–Diagnostic and therapeutic features

**Published:** 2011-05-25

**Authors:** R Costea, E Vasiliu, NO Zarnescu, M Hasouna, S Neagu

**Affiliations:** * ‘Carol Davila’ University of Medicine and Pharmacy, BucharestRomania; ** 2nd Department of Surgery, University Emergency Hospital BucharestRomania

**Keywords:** lipoma–like, liposarcoma, surgery, soft tissue tumor

## Abstract

We are presenting the case of a 44–year old patient with large, well–differentiated liposarcoma of the right thigh. We are discussing the clinical findings, diagnosis and surgical treatment. The large dimensions (27/25 cm) and the origin of the tumor in popliteal fossa, migrating through the adductor canal (Hunter's canal) in the anterolateral muscular space of the right thigh, represent the particularity of this case.

## Introduction

Lipomatous tumors are the most common soft tissue tumors [1]. Over time, major changes in the classification of lipomatous tumors have included the adding of new varieties of lipomas (atypical lipomas), using the term for well–differentiated liposarcoma, and the recognition entity of dedifferentiated liposarcoma. Liposarcomas are mesenchymal tumors that represent the most common type of adult sarcomas, differentiated liposarcomas accounting for 40–50% of all adult liposarcomas [2]. The most frequent locations of lipomatous tumors are the following: the extremities, retroperitoneal, the groin, scrotum, and the abdominal wall [3,4]. Surgical treatment and histology are the most important prognostic factors for patients with lipomatous tumors, as complete surgical excision reduces local recurrence rate [5]. We present a patient with a large lipomatous tumor of the thigh along with diagnostic and therapeutic aspects.

## Case presentation

**Figure 1:**
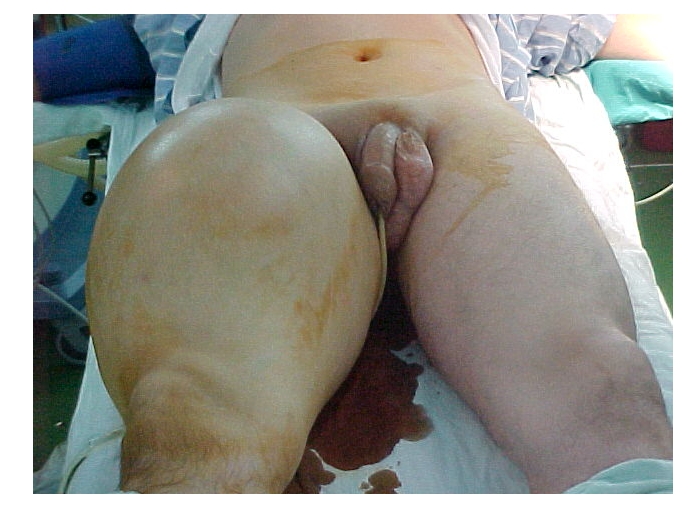
Clinical aspect

A 44 year–old male patient was admitted in our clinic with a giant tumor in the right thigh, which started to grow two years before, with progressive increase in size. The local examination of the right thigh revealed a 35/30 cm high consistency tumor, with reduced mobility. There was no right inguinal adenopathy. (Figure 1)

**Figure 2:**
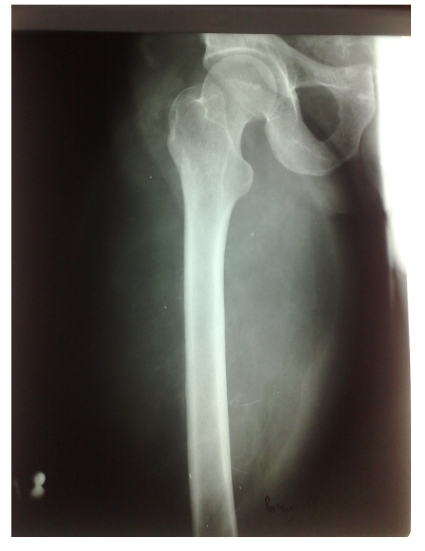
Radiography of right thigh

Blood laboratory tests showed no changes from the normal range. Radiographic examination of the right thigh showed inhomogeneous enlargement of soft parts at this level, with linear opacities. The bone segments did not reveal pathological changes. Ultrasound evaluation showed the formation of a very poorly vascularized giant heterogeneous ultrastructure, which altered the vascular structures of the thigh muscle. (Figure 2)

**Figure 3:**
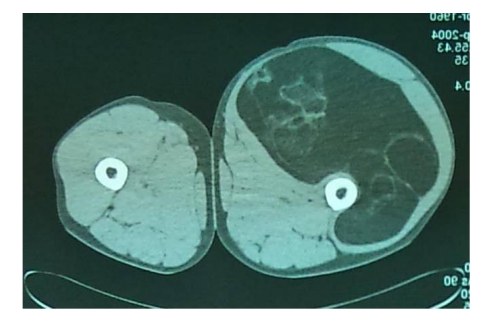
CT scan of the thighs

**Figure 4:**
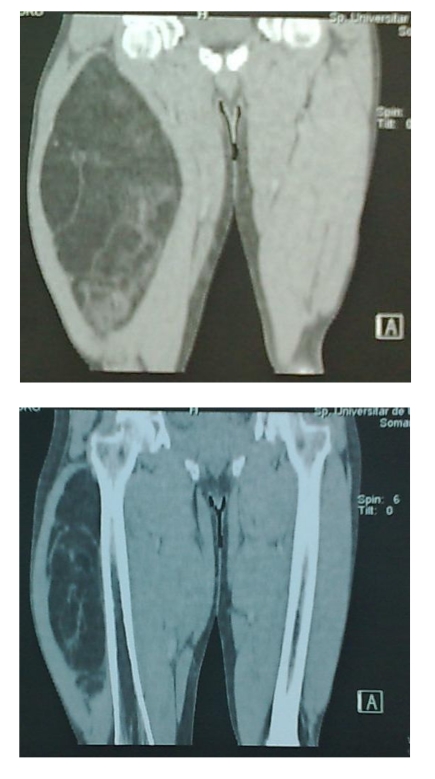
CT scan of the thighs–reconstruction

A CT scan of the right thigh revealed the presence of an expansive process located on the anterior aspect of the thigh starting from the groin to the suprapatellar level, bordered above by sartorius muscle and right femoral muscle and posterior by iliopsoas muscle. Expansive processes measured approximately 11/19 cm (in cross sections), showing that the tumor was well defined, predominantly fatty in density and with numerous irregular septa inside, showing minimal contrast. The femoral artery and vein along with the popliteal vessels showed normal permeability. There were no changes in bone cortex of the right femur and no presence of inguinal lymph nodes. (Figure 3, Figure 4)

**Figure 5:**
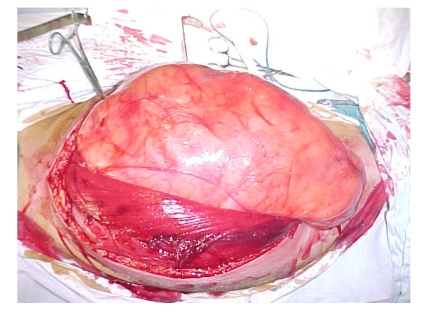
Intraoperator aspect

During surgery, a large, well–marked encapsulated tumor (weight 3,5kg; dimensions 27/25 cm), was found, situated between the adductor muscles and the large muscles that push muscles aside. A simple tumor excision was carried out. (Figure 5)

**Figure 6:**
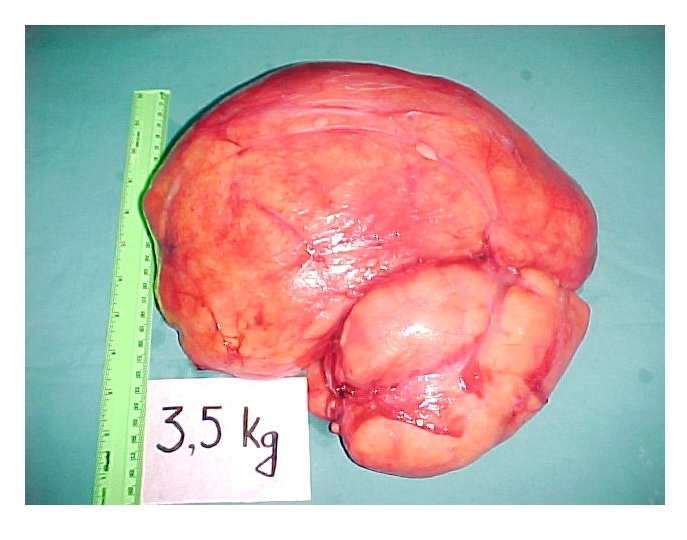
The tumor

**Figure 7:**
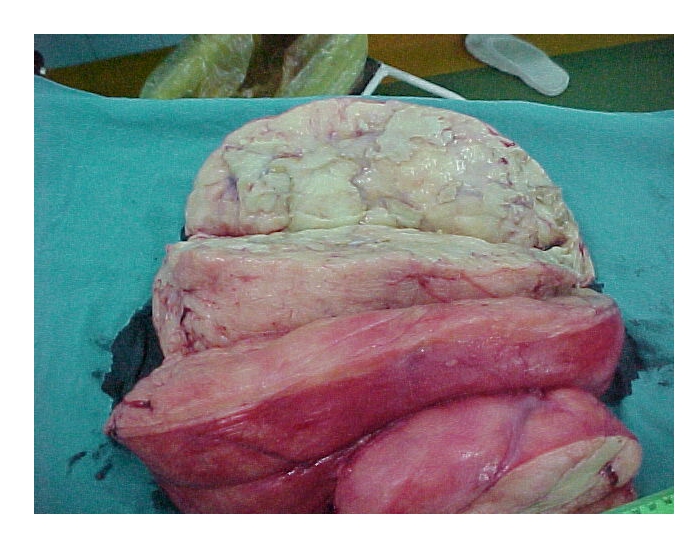
Longitudinal section of the tumor

The pathology exam showed neoplastic proliferation with predominant fat in the form of islands separated by thick well–vascularized conjunctive septa, with areas of tumor necrosis. Adipocytes had nuclei with increased volume and with low pleomorphism. (Figure 6, Figure 7)

Final diagnosis was well–differentiated ‘lipoma–like’ liposarcoma. The patient's state was favorable after the operation, without signs of local or systemic relapse in subsequent follow–up during the next 5 years. The patient did not receive adjuvant radiotherapy or chemotherapy.

## Discussion 

Liposarcomas represent 10–35% of all soft tissue sarcomas, making them the second most common type after malignant fibrous tumors. The WHO classification of soft tissue tumors (2002) divided liposarcomas into five distinct histological subtypes: well differentiated, dedifferentiated, mixoid, pleomorphic and mixed. The diversity of these lesions is reflected by their clinical and biological behavior, which ranges from non–metastatic tumors (well–differentiated liposarcoma) to tumors with high metastatic potential (pleomorphic liposarcoma) [6]. Because of this behavior, pathological and radiological evaluation was critical for establishing an appropriate therapy [7]. 

Supernumerary circular and giant rod chromosomes genetically characterize all subtypes of well–differentiated liposarcomas. These chromosomal abnormalities contain amplification of the 12q13–15 region, which amongst other genes includes MDM2 and CDK4 [8, 9]. Malignant transformation of lipomas has been rarely reported, but, as a rule, well–differentiated liposarcomas are not derived from lipoma [10, 11]. Lipomas do not contain any previously mentioned genetic markers, which is further evidence that well–differentiated liposarcomas are distinct lesions. It was suggested that the implementation of molecular biological analysis of liposarcoma is valuable for adequate classification as a basis for treatment decisions [12].

The image on CT or MRI exams and the morphological relationship between the areas of fat and non-fat components allows the identification of the histological subtype of liposarcomas. Thick connective septa (>2mm) inside of a poorly vascularized fat tumor are suggestive of the diagnosis of well–differentiated liposarcoma [6, 13]. The most difficult differential diagnosis during imaging studies is represented by lipoma [14]. Most lipomas are entirely or predominantly composed of fat with thin septa (<2mm). The right thigh CT scan of our patient revealed the presence of a large tumor with fatty structure, without vascular, bone invasion or enlarged lymph nodes at any level.

Well–differentiated liposarcoma most frequently affects the deep soft tissues of extremities (65–75% of cases); over 50% of these are located in the lower limbs, especially the thigh [15]. Macroscopic appearance of well–differentiated liposarcoma is a large white–yellow well–circumscribed mass. Well–differentiated liposarcoma have no metastatic potential unless dedifferentiation, but they may have local recurrences [16]. Prognosis and treatment of these tumors is strongly related to their anatomical location. For subcutaneously located lesions, surgical excision with wide resection margins is sufficient; in such cases, local recurrence is minimal to nonexistent. For well–differentiated liposarcoma, which are located deeper in the body, the local recurrence risk is high. Studies have shown that local recurrence rate is of 43% for lesions located in the extremities, 70% for groin tumors and 91% for retroperitoneal lesion [17]. The rate of relapse in such cases is high because of the difficulty of obtaining negative surgical margins [18]. In these instances, radiotherapy may be used as adjunctive therapy to decrease local recurrence rate [19]. Mortality associated with multiple complications of local recurrence is significant when well differentiated liposarcoma are located in the retroperitoneal (33%) or inguinal (14%) areas and is insignificant for extremities lesions, if dedifferentiation does not appear in this case [17]. Concerning our patient, there was no muscle, vascular or bone tumor invasion, therefore, despite it s huge size, a large excision allowed an R0 resection (negative surgical margins). Subsequent follow–up did not identify any local recurrence. The absence of local invasion and a lower degree of aggressiveness of the tumor correlates with a favorable prognosis. 

**Figure 8:**
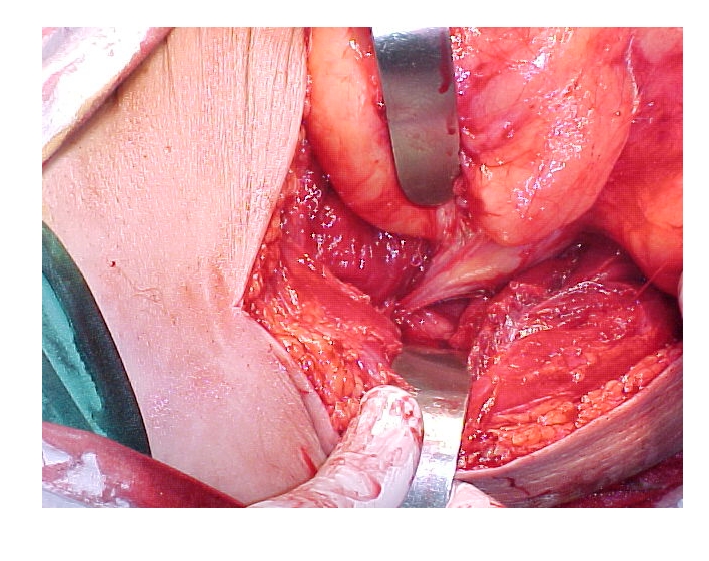
The origin of the tumor in adductor canal (Hunter's canal)

The peculiarity of this case is represented by the large size and the origin of the tumor in popliteal fossa, migrating through the adductor canal (Hunter’s canal) in the anterolateral muscular space of the right thigh. (Figure 8)

## Conclusions

Liposarcoma is one of the most common forms of soft tissue sarcoma, presenting a broad spectrum of clinical behavior. This is closely related to the histological type of liposarcoma and patient management, the evolution being different for each case. Liposarcomas are usually well–differentiated tumors with non-metastatic potential, especially if they are located in the extremities. Understanding and recognizing the broad spectrum of radiological aspects and pathological bases of extremities, liposarcomas allow an improved management of these patients. Despite the huge size that these tumors can reach, large excision decrease local recurrence rate to almost zero. Studies have shown that well–differentiated liposarcoma located on the extremities does not require adjuvant therapy and overall they have prolonged survival and favorable prognosis. 
